# Fosfomycin: A First-Line Oral Therapy for Acute Uncomplicated Cystitis

**DOI:** 10.1155/2016/2082693

**Published:** 2016-05-10

**Authors:** George G. Zhanel, Andrew J. Walkty, James A. Karlowsky

**Affiliations:** Department of Medical Microbiology, College of Medicine, University of Manitoba, Winnipeg, MB, Canada R3A 1R9

## Abstract

Fosfomycin is a new agent to Canada approved for the treatment of acute uncomplicated cystitis (AUC) in adult women infected with susceptible isolates of* E. coli* and* Enterococcus faecalis*. We reviewed the literature regarding the use of oral fosfomycin for the treatment of AUC. All English-language references from 1975 to October 2015 were reviewed. In Canada, fosfomycin tromethamine is manufactured as Monurol® and is available as a 3-gram single dose sachet. Fosfomycin has a unique chemical structure, inhibiting peptidoglycan synthesis at an earlier site compared to *β*-lactams with no cross-resistance with other agents. Fosfomycin displays broad-spectrum activity against ESBL-producing, AmpC-producing, carbapenem-non-susceptible, and multidrug-resistant (MDR)* E. coli*. Resistance to fosfomycin in* E. coli* is rare (<1%). Fosfomycin is excreted unchanged in the urine by glomerular filtration with peak urinary concentration ~4000 *µ*g/mL and remains at concentrations >100 *µ*g/mL for 48 hours after a single 3-gram oral dose. No dosage adjustments are required in elderly patients, in pregnant patients, or in either renal or hepatic impairment. Fosfomycin demonstrates a favorable safety profile, and clinical trials have demonstrated efficacy in AUC that is comparable to ciprofloxacin, nitrofurantoin, and trimethoprim-sulfamethoxazole. Fosfomycin's in vitro activity against common uropathogens, including MDR isolates, its favorable safety profile including pregnancy patients, drug interactions, and clinical trials data demonstrating efficacy in AUC, has resulted in Canadian, US, and European guidelines/authorities recommending fosfomycin as a first line agent for the treatment of AUC.

## 1. Introduction

Urinary tract infections (UTIs) are among the most commonly occurring human infections [1, 2]. It is estimated that approximately 50% of women will experience at least one UTI during their lifetime and that 25% will suffer recurrent infection [3]. Uncomplicated cystitis, the most common presentation for UTI, occurs in adult, premenopausal women with a normal, unobstructed, genitourinary tract where symptoms are confined to the urinary bladder and urethra. Females with cystitis typically present with dysuria and increased urinary urgency and frequency, as well as suprapubic pain, hematuria, and nocturia. Pyelonephritis is distinct from cystitis and is commonly associated with fever (>38°C) and flank pain. The majority of cases of community-acquired cystitis are attributable to uropathogenic* Escherichia coli* (75–90%) and* Staphylococcus saprophyticus* (5–15%), with* Klebsiella* spp.,* Enterococcus* spp.,* Streptococcus agalactiae*, and* Proteus mirabilis* accounting for most other cases [1, 2, 4, 5].* Pseudomonas aeruginosa*,* Staphylococcus aureus*, and* Candida* spp. are infrequent causes of acute uncomplicated UTIs.

Current guidelines published by the Infectious Diseases Society of America (IDSA) and the European Society for Clinical Microbiology and Infectious Diseases (ESCMID) recommend fosfomycin, nitrofurantoin, and trimethoprim-sulfamethoxazole (TMP-SMX) as first-line agents to treat acute uncomplicated UTIs in adult females, reserving fluoroquinolones, amoxicillin-clavulanate, and other *β*-lactams as second-line agents [6]. Elevated rates of resistance (>10–20%) to TMP-SMX, as well as fluoroquinolones, are now widely reported for uropathogenic isolates of* E. coli* in Canada and elsewhere [7, 8]. The most recently published Canadian study, describing antimicrobial resistance rates among* E. coli* isolated from patients with urinary tract infections, reported on isolates collected from 2010 to 2013 and found susceptibility rates of 74.7% to TMP-SMX, 77.4% to ciprofloxacin, 81.3% to amoxicillin-clavulanate, 96.1% to nitrofurantoin, and 99.4% to fosfomycin [9]. The increasing identification of extended-spectrum beta-lactamase- (ESBL-) producing* E. coli* across Canada and internationally has been associated with concomitant resistance to amoxicillin-clavulanate, ciprofloxacin, and TMP-SMX [7, 8]. Rates of susceptibility among* E. coli* of <80% for one or more first- or second-line agents should prompt local reevaluation of empiric treatment strategies for acute uncomplicated UTIs [6].

This review endeavoured to summarize peer-reviewed published data on the development of fosfomycin, its chemistry, mechanisms of action and resistance, in vitro microbiology, pharmacokinetic and pharmacodynamic properties, efficacy demonstrated in clinical trials for acute cystitis, adverse effects, drug interactions, and its role in therapy of acute cystitis.

## 2. Development of Fosfomycin

In 1969, laboratories at MSD (Merck, Sharp, and Dohme) and CEPA (Compañia Española de Penicilina y Antibioticos) were the first to successfully isolate fosfomycin, a phosphonic acid derivative, from cultures of* Streptomyces* spp. (*S. fradiae*,* S. viridochromogenes*, and* S. wedomorensis*) [10, 11]. Fosfomycin was initially synthesized as a calcium salt [11]; however, this formulation was later identified to be responsible for discrepancies reported by in vitro studies and for delayed verification of therapeutic effectiveness in animal models and patients, and its commercial production was discontinued. Fosfomycin was subsequently reformulated into a new salt, fosfomycin tromethamine, also known as fosfomycin trometamol. Fosfomycin tromethamine demonstrated the same spectrum of activity and safety profile as the calcium salt as well as producing improved oral bioavailability and consistency for in vitro antimicrobial susceptibility testing [12, 13].

Fosfomycin has been available to physicians in many European countries as well as Japan, South Africa, and Brazil, in both oral and parenteral formulations, for up to four decades [12, 13]. Parenteral fosfomycin is formulated as a disodium salt. Oral fosfomycin first entered the Canadian and American markets in 1997 [3] but was withdrawn in Canada several years later due to lack of use. It was recently reintroduced in Canada and has been added to several provincial formularies with an indication for the treatment of acute uncomplicated cystitis in adult women infected with susceptible isolates of* E. coli* and* Enterococcus faecalis* [14]. In Canada and the United States, fosfomycin tromethamine is manufactured exclusively under the brand name Monurol and is available as a 5.7-gram powder sachet of which 3 grams is fosfomycin [14].

## 3. Chemistry

**Figure 1:**
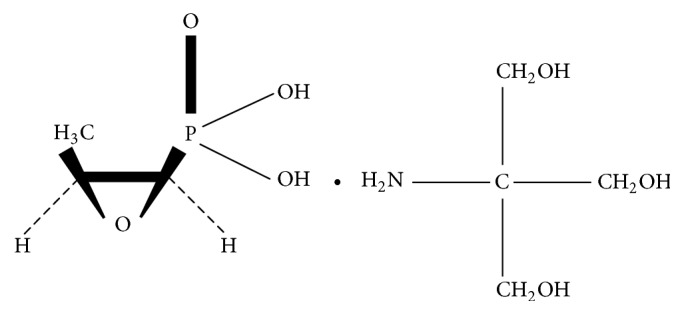
Chemical structure of fosfomycin tromethamine.

The chemical structure of fosfomycin ([−] [1R, 2S]-1,2-eposipropylphosphonic acid or cis-1,2-epoxypropyl phosphoric acid) was published in 1969 shortly after its initial isolation [10] (Figure 1). It has a molecular mass of 138.059 g/mol (C_3_H_7_O_4_P). The structure of fosfomycin has two distinguishing features, a stable epoxide group and a phosphonic acid moiety; both are key components of its therapeutic activity. Its carbon-phosphorous bond is also unique to a minority of naturally occurring compounds and is an indication that it is the product of a distinct and complex biosynthetic process. The biosynthetic pathway of fosfomycin was initially described in* S. wedomorensis* and leads to the development of an in vitro chemical synthesis process, using phosphonic acid as starting material, which is currently used in commercial production [15].

## 4. Mechanism of Action

Fosfomycin's mechanism of action results from its irreversible inhibition of MurA (UDP-N-acetylglucosamine-3-enolpyruvyl transferase), the cytosolic enzyme responsible for the first step in the peptidoglycan biosynthesis pathway that produces UDP-N-acetylmuramic acid [16]. More specifically, fosfomycin is a phosphoenolpyruvate (PEP) analog. MurA is responsible for ligating PEP to the 3′-hydroxyl group of UDP-N-acetylglucosamine in the pathway that produces UDP-N-acetylmuramic acid [16]. The inhibition of MurA is the result of direct nucleophilic attack by a catalytic site cysteine residue on the C-2 carbon of fosfomycin, resulting in blockage of the catalytic site [16]. Fosfomycin has minimal to no effect on other enzymes utilizing PEP, such as enolase, pyruvate kinase, and PEP carboxykinase [16]. Fosfomycin's mechanism of action is unique and distinct from other bacterial cell wall inhibitors (*β*-lactams and glycopeptides) as well as other classes of antibacterial agents suggesting that the likelihood of cross-resistance to these other agents should be minimal.

Fosfomycin enters the bacterial cytosol by two transport systems, the constitutively expressed L-*α*-glycerophosphate (or glycerol-3-phosphate) uptake (GlpT) system and the glucose-6-phosphate- (G6P-) inducible hexose-monophosphate transport (UhpT) system. Of the two systems, the G6P-inducible UhpT system serves as the primary portal of entry for fosfomycin [16]. In 1983, Andrews and coworkers [17] demonstrated that a G6P concentration of 25 *μ*g/mL optimizes induction of the UhpT system and it was subsequently added to standardized in vitro susceptibility testing methods for fosfomycin to facilitate reproducible MIC generation [18]. Most Enterobacteriaceae (excluding* Proteus* spp.),* Enterococcus* spp., and* Staphylococcus* spp. possess the UhpT transport system in their cell membrane [16, 17].

## 5. Mechanisms of Resistance

In vitro resistance to fosfomycin has been associated most commonly with chromosomal mutations in GlpT and less frequently with mutations in UhpT [16, 19–22]. However, the presence of a functional G6P-inducible UhpT transport system frequently overrides resistance produced by mutations in GlpT and results in a fosfomycin susceptible phenotype [16]. Resistance to fosfomycin may also result less commonly from a myriad of other mechanisms including modification, inactivation, or overexpression of MurA, fosfomycin kinases, or the inactivation via plasmid-mediated enzymes such as* fosA*,* fosB*,* fosC*, and* fosX* [23].

Plasmid-mediated* fos* enzymes are members of the glyoxalase superfamily and inactivate fosfomycin by catalyzing its conjugation with glutathione or another nucleophile. These enzymes function by nucleophilic attack on carbon 1 of fosfomycin, which opens the epoxide ring and inactivates it. The* fos* enzymes differ by the identity of the nucleophile utilized in the reaction: glutathione is used by FosA, bacillithiol by FosB, and water by FosX [21, 24, 25]. In general, FosA and FosX enzymes are produced by Gram-negative bacteria and FosB is produced by Gram-positive bacteria [21]. Another enzyme, FosC, utilizes ATP and adds a phosphate group to fosfomycin, which also neutralizes its antibacterial properties [26].

Resistance development during therapy is a confounding issue for fosfomycin. In vitro studies have shown that fosfomycin can be associated with the development of resistance at a frequency of 10^−8^ to 10^−6^ [23, 27–29]. However, the frequency of mutational resistance in vitro is not observed in clinical studies suggesting that there may be a biological cost associated with common mutations that confer resistance to fosfomycin in vitro [22, 23]. Experimental studies with fosfomycin-resistant* E. coli* isolates have also demonstrated reduced epithelial cell adherence, increased susceptibility to polymorphonuclear cell and serum complement killing, and slower growth rates [12, 22, 30]. Overall, declines in bacterial virulence associated with fosfomycin-resistant* E. coli* may explain the low rates of resistance observed in vivo for this agent despite decades of use [12]. Data from the Antimicrobial Resistance Epidemiological Survey on Cystitis study showed that resistance to fosfomycin remains rare in regions where it is widely used (~2%) [31]. Other reasons for fosfomycin's low resistance rate in urinary tract infections may include its short contact time, high urine concentration, and potentially higher compliance compared with agents dosed for 3–7 days. Lower frequencies of resistance development have been observed at higher fosfomycin concentrations and in media with an acidic pH [23]. In vitro resistance development in* E. coli* is less frequent than in* K. pneumoniae* and* P. aeruginosa* [23]. Although the limited resistance in* E. coli* to fosfomycin from a variety of regions across the world (despite intensive use of this agent) is encouraging, we need to remain vigilant with ongoing surveillance studies to assess susceptibility to fosfomycin in Canada. Most worrisome would be the emergence of plasmid-mediated enzymes such as* fosA*,* fosB*,* fosC*, and* fosX* [23].

## 6. In Vitro Microbiology

Standardized methods for antimicrobial susceptibility testing of fosfomycin are published by the Clinical and Laboratory Standards Institute (CLSI) [18] and the European Committee on Antimicrobial Susceptibility Testing (EUCAST) [32]. It is important to recognize that differences exist between these two standards. Specifically, there are differences in the organisms for which fosfomycin MIC and zone diameter breakpoints apply in the two standards as well as numerical differences in fosfomycin MIC and zone diameter breakpoints.

Currently, CLSI-approved agar dilution susceptibility breakpoints for fosfomycin exist only for* E. coli* and* E. faecalis*, with a MIC ≤64 *μ*g/mL considered susceptible (resistance, ≥256 *μ*g/mL), and are approved only for testing isolates from urinary tract infections [18]. EUCAST breakpoints for fosfomycin apply to all Enterobacteriaceae with a MIC ≤32 *μ*g/mL considered susceptible (resistance, >32 *μ*g/mL) for both oral (uncomplicated urinary tract infection only) and parenteral (systemic infections) fosfomycin [32]. EUCAST also publishes parenteral fosfomycin breakpoints for staphylococci but no breakpoints for enterococci. Based on our own data with* E. coli*, the difference in fosfomycin susceptibility using CLSI or EUCAST breakpoints is <1% [9].

Antimicrobial susceptibility testing (agar dilution or disk diffusion) requires agar supplementation with 25 *μ*g/mL of G6P to ensure induction of the UhpT pathway [18, 32]. Studies reporting in vitro susceptibility testing of fosfomycin prior to 1983 should be disregarded as the importance of adding physiological levels of G6P to testing media was unknown before that time [12, 17]. Further, studies based upon broth dilution MIC testing should not be considered because of the relatively high likelihood of spontaneous mutation to fosfomycin resistance in broth [12]. Presently, we are not aware of efforts by automated susceptibility systems such as Vitek in testing fosfomycin in Canada or the US, but these efforts are required.

In general, fosfomycin demonstrates moderate in vitro potency against both Gram-negative and Gram-positive bacterial pathogens, including those most commonly associated with cystitis (*E. coli*,* Klebsiella* spp.,* Enterococcus* spp., and* Proteus* spp., but not* S. saprophyticus*) [9, 12, 32–42].

Table 1 presents the in vitro activity of fosfomycin against aerobic and facultative Gram-negative bacteria from various specimen sources, including urine isolates. In all studies, MICs were determined using either the CLSI [18] or EUCAST [32] standard method. Fosfomycin has higher MICs for* Klebsiella* spp.,* Enterobacter* spp., and* Serratia* spp., than for* E. coli*,* Citrobacter* spp., and* Proteus* spp. The activity of fosfomycin against* Klebsiella* spp. and* Enterobacter* spp. demonstrates some variability. Fosfomycin may be active against some isolates of* P. aeruginosa* with MICs ranging from 4 to >512 *μ*g/mL.* Acinetobacter baumannii* appear inherently resistant to fosfomycin. However, studies combining fosfomycin with other agents such as cefepime and meropenem versus* A. baumannii* or an aminoglycoside for* P. aeruginosa* have demonstrated additivity between the two agents [43]. Gram-negative anaerobic bacteria have been reported to not be a part of fosfomycin's antibacterial spectrum [12] but the reasons for this are cryptic.

Fosfomycin retains its in vitro activity against ESBL-producing, AmpC-producing, carbapenem-non-susceptible, and multidrug-resistant (MDR)* E. coli*, as well as KPC-producing* K. pneumoniae* [9, 38–41, 44]. Kaase et al. tested 80 isolates of Enterobacteriaceae with various carbapenemases (KPC, VIM, NDM, and OXA-48) and reported that 78% had MICs ≤32 *μ*g/mL and would thus be considered susceptible according to the EUCAST breakpoint [45]. In another study, Falagas and others tested 152 MDR Enterobacteriaceae (76%* K. pneumoniae*, 17%* E. coli*, 5%* P. mirabilis*, and 2% others) and determined that 93% of isolates were susceptible to fosfomycin by CLSI breakpoints (MIC, ≤64 *μ*g/mL) [46]. Subgroup analysis in the Falagas et al. study showed that 95%, 94%, and 83% of carbapenemase-producing (*n* = 79), ESBL-producing (*n* = 34), and metallo-*β*-lactamase-producing (*n* = 24) isolates were susceptible to fosfomycin [46].

Table 2 summarizes data presented in the most recently published Canada-wide surveillance study of* E. coli* isolates collected from patients with UTIs from 2010 to 2013 [9]. Rates of susceptibility to fosfomycin were 99.4%, 97.9%, 99.1%, 100%, 100%, and 100% for all isolates, ciprofloxacin-resistant, TMP-SMX-resistant, ESBL-producing, AmpC-producing, and MDR isolates, respectively, superior to nitrofurantoin and other frequently prescribed oral empiric agents [9].

The in vitro activity of fosfomycin against Gram-positive bacteria from various specimen sources, including urine isolates, is summarized in Table 3. Fosfomycin appears more active in vitro against* S. aureus*, including methicillin-resistant* S. aureus* (MRSA), and* S. pneumoniae* than other Gram-positive bacteria. The majority (>50%) of isolates of* S. aureus*, enterococci (including vancomycin-resistant enterococci (VRE)), and streptococci have fosfomycin MICs ≤32 *μ*g/mL. Some streptococci,* Staphylococcus saprophyticus*, corynebacteria,* Chlamydia*, and mycoplasmas are resistant to fosfomycin likely due to the absence or low abundance of the G6P-inducible UhpT system or an altered MurA target [12, 47].

## 7. Pharmacokinetic Properties

Fosfomycin is freely soluble in water, demonstrates negligible plasma protein binding, and distributes widely into tissues (steady state volume of distribution is 136.1 ± 44.1 L) [12, 14, 23, 27, 43, 48–50].

Table 4 summarizes the pharmacokinetic properties for fosfomycin tromethamine following oral administration of a single 5.7-gram (3-gram fosfomycin) dose. The oral bioavailability of fosfomycin is 34–41%, 18% is recovered from feces, and 54–65% of absorbed fosfomycin is recovered unaltered in the urine [14, 48].

Fosfomycin's distribution follows a two-compartment model [48, 51]. Upon absorption from the gut, fosfomycin is rapidly distributed to the kidneys, bladder, prostate, and seminal vesicles [14, 49]. A 3-gram oral dose of fosfomycin results in serum *C*_max_ of 22–32 *µ*g/mL achieved within 2–2.5 hours. Animal studies have demonstrated that fosfomycin penetrates fluids and tissue of the central nervous system and cardiac, respiratory, and urinary systems supporting its use in noncystitis indications such as prostatitis [52].

Fosfomycin is not metabolized but rather is excreted unchanged in the urine by glomerular filtration [14, 49]. Its serum half-life is 5.7 hours [12, 14]. Peak urinary concentrations reach ~4000 *µ*g/mL and remain at concentrations >100 *µ*g/mL for 48 hours [49]. A fosfomycin urinary concentration above the MIC_90_ of* E. coli* (4 *µ*g/mL) has been reported for 80 hours [48].

No dosage adjustments are required in elderly patients, pregnant patients, or either renal or hepatic impairment [14, 48, 49]. Studies have reported an increase in *T*_max_ and *C*_max_, larger AUC, and reduced rates of elimination for renally impaired patients; regardless, no dosage adjustment is recommended for patients with a creatinine clearance (Cr_CL_) >10 mL/min [14, 48, 49]. A study of patients with (Cr_CL_) of 7–54 mL/min demonstrated increased serum *t*_1/2_ from 11 h to 50 h, accompanied by the observation that only 30% of orally absorbed fosfomycin was excreted within 24 hours [49]. Another study of patients with mean Cr_CL_ of 40 mL/min demonstrated lower mean urinary concentrations for the first 24 hours, but similar concentrations after 48–60 hours [14]. Limited data exists for both children and pregnant women. Studies have demonstrated slightly higher elimination rates in children, as well as an increase in mean urinary fosfomycin concentrations in pregnant patients, although the differences were considered insignificant and did not warrant dose adjustments [14].

## 8. Pharmacodynamic Properties

Fosfomycin has demonstrated concentration-dependent killing in two different in vitro models. Mazzei and colleagues assessed fosfomycin activity using kill curves at concentrations from 1x MIC to 64x MIC for isolates of* E. coli* and* P. mirabilis* [53]. When bacterial growth was assessed from time 0 to 24 hours, bacterial inhibition was directly proportional to fosfomycin concentration [53]. For* E. coli*, complete eradication was observed at 6–8 hours at fosfomycin concentrations ≥4x MIC [53].

In a hollow fiber infection model that simulated human pharmacokinetics of fosfomycin, three isolates of ESBL-producing* E. coli* (Ec2974: fosfomycin MICs 1 *µ*g/mL, Ec46: MIC 1 *µ*g/mL, and Ec4244: MIC 64 *µ*g/mL, resp.) were challenged with varying dosage regimens to assess bacterial inhibition, including 4 g q8h (12 g/day), 8 g q8h (24 g/day), and 12 g q8h (36 g/day), and concentration-dependent activity was observed [54]. Fosfomycin *f*AUC_0–24_/MIC ratios (MIC, 1 *µ*g/mL) were determined to be 1,744.94, 3,3136.03, and 4,287.82 for 12 g/day, 24 g/day, and 36 g/day doses, respectively [54]. While growth of highly resistant mutants in the 12 g/day dose was observed after 24 hours, higher dosages (24 g/day and 36 g/day) resulted in complete bacterial eradication and suppression of resistant mutants [54]. Furthermore, no difference in the rate and extent of fosfomycin's bactericidal activity was observed with different dosage intervals suggesting that fosfomycin's pharmacodynamic activity was not time-dependent [54].

The postantibiotic effect (PAE) for fosfomycin has also been assessed [53]. Following two-hour exposures to varying concentrations of fosfomycin (0.25x MIC, 1x MIC, 4x MIC, and 8x MIC) and subsequent removal,* E. coli* and* P. mirabilis* demonstrated long, concentration-dependent PAEs ranging from 3.4 hours at 0.25x MIC to 3.4 hours at 1x MIC to 4.2 h at 8x MIC [49, 53].

## 9. Clinical Trials

In 2010, Falagas and others published a meta-analysis of all available, randomized controlled trials comparing fosfomycin and other antimicrobial agents for effectiveness and safety in the treatment of acute uncomplicated cystitis [46]. Following screening, they included 27 trials from 1987 to January 2010 in their analysis [46]. They reported no difference in clinical success, microbiological success, relapse, or reinfection with single dose fosfomycin and comparators using multidose regimens in trials involving nonpregnant females (*n* = 16 trials) and trials involving adult mixed populations (*n* = 3 trials). They were unable to draw conclusions from trials involving pediatric patients (*n* = 3 trials) and pregnant patients (*n* = 5 trials) because of insufficient data. Fosfomycin was shown to possess a comparable safety profile with the comparators prescribed for nonpregnant women, adult mixed populations, and pediatric patients and to have a significantly lower rate of adverse events in pregnant women [46]. The authors concluded that fosfomycin may provide a valuable alternative therapy for the treatment of acute cystitis in nonpregnant and pregnant women, as well as in the elderly and pediatric patients.

To further the work of Falagas et al., we performed a review of randomized, controlled trials from 2010 to 2015 that compared fosfomycin and other antimicrobial agents for the treatment of acute uncomplicated cystitis and one additional study was identified. Ceran and colleagues conducted a randomized comparative study of single-dose fosfomycin and 5-day ciprofloxacin in 260 female patients, aged between 18 and 65 years, with uncomplicated lower urinary tract infections [55]. Of the 260 patients enrolled, 142 completed the study. In the 142 patients,* E. coli* (82.3%) and* Enterobacter* spp. (8.4%) were the pathogens most frequently isolated from urine cultures. Patients were evaluated on days 7–10 for clinical and microbiological cure. Clinical cure rates were 83% for fosfomycin and 81% for ciprofloxacin; microbiological cure rates were 83% for fosfomycin and 78% for ciprofloxacin. Patients were reevaluated on day 60 for recurrence or reinfection; only five patients of 142 patients demonstrated relapse. The authors concluded that a single 3-gram dose of fosfomycin was as effective as ciprofloxacin, at 500 mg twice a day for 5 days, in the treatment of acute uncomplicated cystitis.

## 10. Adverse Effects

Most adverse effects described for fosfomycin have been mild and transient [48].  Table 5 summarizes the most commonly reported adverse effects of fosfomycin. Serious adverse reactions such as angioedema, asthma exacerbation, cholestatic jaundice, hepatic necrosis, and toxic megacolon have been extremely rare [48]. As with other antimicrobial agents, prolonged use of fosfomycin may be associated with fungal or bacterial super-infections, including* Clostridium difficile* infection [47]. A postmarketing study in Japan (reporting on fosfomycin calcium) documented only 2 cases of super-infection within 6 years [56]. Laboratory changes (including increased eosinophil count, increased or decreased white blood cell count, increased bilirubin, increased alanine aminotransferase, increased aspartate aminotransferase, increased alkaline phosphatase, decreased hematocrit, decreased hemoglobin, and alterations in platelet count) have also been reported; however, they have been transient and clinically insignificant [47].

## 11. Drug Interactions

The only confirmed drug interaction for fosfomycin is with metoclopramide [14, 47, 49, 57]. Metoclopramide's mechanism of action (increase in gastrointestinal mobility) decreases the absorption of fosfomycin tromethamine, resulting in a 25% reduction in *C*_max_ and 20-minute shorter *t*_max_ [47, 48]. Recent data also suggest that fosfomycin may have a protective effect against nephrotoxicity induced by aminoglycosides or cisplatin, which may be due to fosfomycin's inhibition of medication-induced histamine release from mast cells [56]. Lastly, no drug interactions have been reported between fosfomycin and commonly used agents such as analgesics, anticonvulsants, bronchodilators, diuretics, spasmolytics, and antipyretics [14].

## 12. Role of Fosfomycin in Therapy of Acute Cystitis

In vitro studies have demonstrated increasing rates of resistance among* E. coli* urinary isolates to trimethoprim-sulfamethoxazole and ciprofloxacin, antimicrobial agents frequently prescribed for the treatment of cystitis [7, 57, 58]. Community-acquired UTIs caused by ESBL-producing* E. coli* are also being reported with increasing frequency [59]. These isolates are often MDR, leaving clinicians with few oral treatment options [59]. Fosfomycin demonstrates excellent in vitro activity against common uropathogens, including MDR isolates [9]. Fosfomycin demonstrates a favorable safety profile, and clinical trials have demonstrated efficacy in the treatment of uncomplicated cystitis that is comparable to other first-line antimicrobials [46, 48, 60]. Fosfomycin has a unique chemical structure that is distinct from all other marketed antimicrobial classes (i.e., *β*-lactams, glycopeptides, fluoroquinolones, macrolides, lincosamides, tetracyclines, aminoglycosides, etc.) and there is no cross-resistance with other agents used to treat cystitis [12]. Resistance to fosfomycin most commonly arises by chromosomal mutations which do not spread easily, and the biological cost of these genetic mutations is high [22, 23]. As an additional advantage, when used for the treatment of acute uncomplicated cystitis, fosfomycin is conveniently administered as a single 3 g oral dose [6]. Current UTI guidelines from the Infectious Diseases Society of America, the European Society for Microbiology and Infectious Diseases, the European Association of Urology, and the Canadian Anti-Infective Guidelines for Community Acquired Infections (the Ontario Orange Book) all recommend fosfomycin as a first-line antimicrobial for the treatment of acute, uncomplicated cystitis [6, 60–62]. This recommendation is supported by the data reviewed here. Given the rising antimicrobial resistance rates among common uropathogens, it is likely that fosfomycin will be used with increasing frequency over the coming years.

## Figures and Tables

**Table 1:** In vitro activity of fosfomycin against aerobic and facultative Gram-negative bacteria^a^.

Bacteria	Number tested	Fosfomycin		
		MIC_50_ (*μ*g/mL)	MIC_90_ (*μ*g/mL)	Range (*μ*g/mL)
*Acinetobacter *spp.	185	64–128	128–512	0.25–512
*Citrobacter* spp. (*C. diversus* and* C. freundii*)	251	1-2	4–16	0.03–128
*Enterobacter *spp. (*E. agglomerans, E. aerogenes,* and *E. cloacae*)	779	8–64	32–256	0.12–>512
*Escherichia coli*	9338	0.5–4	2–16	0.03–512
*Escherichia coli* ESBL-producing	362	0.5–2	2–32	0.03–512
*Escherichia coli* AmpC-producing	135	2	4–16	≤1–>512
*Haemophilus influenzae*	50	1	4	1–128
*Klebsiella oxytoca*	51	8	32	4–64
*Klebsiella pneumoniae*	392	16–32	32–>128	0.5–512
*Klebsiella pneumoniae* ESBL-producing	74	16	64	2–256
*Klebsiella* spp.	995	8–16	32–128	≤2–512
*Morganella morganii*	98	128–256	512	8–>512
*Proteus mirabilis*	1472	≤2–4	32–>128	≤1–>512
*Proteus vulgaris* (indole-positive *Proteus*)	341	≤2–16	8–256	≤2–256
*Providencia* spp. (*P. rettgeri *and* P. stuartii*)	164	2–16	8–128	≤2–512
*Pseudomonas aeruginosa*	1518	32–256	64–256	4–>512
*Pseudomonas *spp.	35	128	256	≤0.5–512
*Serratia marcescens*	307	8–16	16–128	≤2–128
*Shigella* spp.	185	2	2	0.5–64
*Stenotrophomonas maltophilia*	49	64	128	16–512

^a^Data compiled from [9, 12, 32–41].

AmpC, chromosomal AmpC *β*-lactamase; ESBL, extended-spectrum *β*-lactamase.

MIC_50_,  minimum concentration (*μ*g/mL) required to inhibit the growth of 50% of isolates; MIC_90_, minimum concentration (*μ*g/mL) required to inhibit the growth of 90% of isolates.

**Table 2:** In vitro activities of orally prescribed antimicrobial agents against *E. coli* isolated from urine specimens in clinical laboratories across Canada from 2010 to 2013^a^.

*E. coli* isolate phenotype^b^ (number tested)	Antimicrobial agent	MIC interpretation	
		% susceptible	% resistant
All *E. coli* (868)	Fosfomycin	99.4	0.1
	Amoxicillin-clavulanate	81.3	5.7
	Ciprofloxacin	77.4	22.5
	Nitrofurantoin	96.1	1.5
	TMP-SMX^c^	74.7	25.3

TMP-SMX-resistant (219)	Fosfomycin	99.1	0
	Amoxicillin-clavulanate	67.1	6.4
	Ciprofloxacin	51.6	47.9
	Nitrofurantoin	91.8	3.2
	TMP-SMX	0	100

Ciprofloxacin-resistant (195)	Fosfomycin	97.9	0
	Amoxicillin-clavulanate	66.0	6.7
	Ciprofloxacin	0	100
	Nitrofurantoin	91.3	4.1
	TMP-SMX	45.9	54.1

ESBL-producing (42)	Fosfomycin	100	0
	Amoxicillin-clavulanate	33.3	11.9
	Ciprofloxacin	9.5	90.5
	Nitrofurantoin	83.3	4.8
	TMP-SMX	35.7	64.3

AmpC-producing (16)	Fosfomycin	100	0
	Amoxicillin-clavulanate	6.3	87.4
	Ciprofloxacin	75.0	25.0
	Nitrofurantoin	100	0
	TMP-SMX	75.0	25.0

Multidrug-resistant (15)	Fosfomycin	100	0
	Amoxicillin-clavulanate	13.3	66.7
	Ciprofloxacin	0	100
	Nitrofurantoin	60.0	40.0
	TMP-SMX	6.7	93.3

^a^Data adapted from [9].

^b^ESBL, extended-spectrum *β*-lactamase; AmpC, chromosomal AmpC *β*-lactamase; multidrug-resistant was defined as isolates resistant to ≥3 agents from different antimicrobial classes (amoxicillin-clavulanate, ciprofloxacin, nitrofurantoin, and TMP-SMX).

^c^TMP-SMX, trimethoprim-sulfamethoxazole.

**Table 3:** In vitro activity of fosfomycin against facultative Gram-positive bacteria^a^.

Organism	Number tested	Fosfomycin		
		MIC_50_ (*μ*g/mL)	MIC_90_ (*μ*g/mL)	Range (*μ*g/mL)
*Enterococcus faecalis*	1862	32	64	0.5–512
*Enterococcus faecium*	516	32–64	64–128	0.5–128
*Enterococcus* spp.	137	16–32	64	0.25–>256
*Staphylococcus aureus*	2213	4	16	0.12–512
*Staphylococcus aureus*, MRSA	162	4	64	0.5–512
*Staphylococcus epidermidis*	896	8	128	0.5–256
*Staphylococcus saprophyticus*	227	64–128	256–>512	≤2–>512
*Streptococcus pneumoniae*	57	8	16	4–32
*Streptococcus pyogenes*	50	32	64	2–64
*Streptococcus agalactiae*	50	32	64	2–64

^a^Data compiled from [12, 32–34, 38, 42].

**Table 4:** Pharmacokinetic properties of fosfomycin following a single 3-gram oral dose^a^.

Parameter	Mean value or range
Serum/plasma	
Bioavailability (*F*)	34–41%
Maximum plasma concentration *C*_max_	22–32 *μ*g/mL
Time to maximum concentration in the blood (*T*_max_)	2–2.5 h
Area under the curve (AUC)	145 mg/L·h
Volume of distribution (*V*_*d*_)	136.1 L
Half-life (*t*_1/2_)	5.7 h
Clearance (CL)	16.9 L/h

Urine	
Maximum urinary concentration (*U*_max_)	1053–4415 *μ*g/mL
Time to maximum concentration in the urine (urinary *t*_max_)	4 h
Urinary concentration at 48 h	~100 *μ*g/mL

Dosage adjustments	
Dose adjustment in elderly	None required
Dose adjustment in pregnancy	None required
Dose adjustment for renal impairment	None required
Hepatic adjustment	None required

^a^Data adapted from [14, 48, 49].

**Table 5:** Adverse effects of fosfomycin following a single 3-gram oral dose^a^.

Adverse effect	Frequency (%)
Diarrhea	4–10
Vaginitis	5.8
Headache	2
Nausea	2–5
Epigastric/abdominal pain	1.3–2
Dyspepsia	1-2
Dizziness	1.2
Asthenia	1
Fatigue	0.2
Mild backache	0.2

^a^Data adapted from [47, 48, 56].
